# Functional interaction between orexin/dynorphin transmission in the posterior paraventricular nucleus of the thalamus following alcohol dependence: mediation of alcohol-seeking behavior

**DOI:** 10.3389/fphar.2025.1718540

**Published:** 2025-11-27

**Authors:** Francisco J. Flores-Ramirez, Glenn Pascasio, Rémi Martin-Fardon

**Affiliations:** 1 Department of Translational Medicine, The Scripps Research Institute, La Jolla, CA, United States; 2 Department of Psychology, California State University San Marcos, San Marcos, CA, United States

**Keywords:** alcohol dependence, alcohol-seeking behavior, orexin (hypocretin), dynorphin, orexin receptors 1 and 2, kappa opioid receptor (KOR)

## Abstract

Stress is a major contributor to the chronic nature of alcohol use disorder (AUD). Orexin (OX) neurons project to the paraventricular nucleus of the thalamus (PVT)—particularly the posterior part (pPVT)— a structure that plays a key role in stress regulation. The blockade of OX receptors in the pPVT was shown to prevent the stress-induced reinstatement of alcohol seeking in alcohol-dependent rats. Accumulating evidence indicates interactions among OX and dynorphin (DYN) in the pPVT, but unclear is the role of OX and DYN transmission in the pPVT in the stress-induced alcohol seeking during alcohol abstinence. Male Wistar rats were trained to self-administer 10% alcohol for 3 weeks. They then underwent 6 weeks of chronic intermittent alcohol vapor exposure to induce dependence. After 12 extinction sessions (∼3 weeks of abstinence), the rats received intra-pPVT infusions of the dual OX receptor antagonist TCS1102 (15 µg/0.5 µL), the κ-opioid receptor (KOP) antagonist nor-binaltorphimine (norBNI; 4 µg/0.5 µL), or their combination, and they were assessed for their reactivity to the stress (footshock)-induced reinstatement of alcohol-seeking behavior. In dependent rats, TCS1102 and norBNI reduced reinstatement but, when co-administered, their individual effects were modulated. At the time of testing, increases in *Hcrt* and *Pdyn* mRNA expression in the hypothalamus and a decrease in *Hcrtr1* expression and an increase in *Oprk1* expression in the pPVT were observed. These findings reveal a functional interaction among OX receptor and KOP signaling in the pPVT that underlies relapse that is precipitated by stress post-dependence, underscoring the value of multi-target interventions to restore pPVT function and prevent relapse.

## Introduction

Alcohol use disorder (AUD) is characterized by its significant contribution to global disability and preventable death and is one of the most widespread mental health conditions, imposing substantial public health, economic, and social burdens (Grant et al., 2015; Grant et al., 2004; MacKillop et al., 2022; Rehm et al., 2015; Witkiewitz et al., 2019; Yaseen et al., 2024). Despite notable therapeutic advances for chronic illnesses like metabolic disorders, cardiovascular disease, and autoimmune conditions, clinicians still lack reliably safe and effective treatments for AUD (Pierce et al., 2012). One core obstacle in AUD management is the high rate of relapse to drinking, even after extended periods of voluntary abstinence. Stress, described as any disturbance to physiological homeostasis, is widely recognized as a critical factor that underlies the chronic, relapsing, and compulsive nature of alcohol dependence. This stems from the ability of stress to provoke intense craving during recovery from AUD and its ability to trigger alcohol-seeking behavior (Sinha, 2024; Stephens and Wand, 2012). Indeed, research consistently shows that individuals who face high social stressors relapse at higher rates post-treatment compared with those who experience less stress (Brown et al., 1990; Noone et al., 1999).

Stress significantly increases the vulnerability to AUD and relapse, but the exact reasons behind this have not been fully elucidated. The body’s main stress response system (i.e., the hypothalamic-pituitary-adrenal [HPA] axis) is believed to play a major role (Wand, 2008). Alcohol initially causes pleasant effects, including stimulation and relaxation, at low doses (Knight et al., 2020), but repeated cycles of heavy alcohol use disrupt how the HPA axis handles stress. As dependence develops, these disruptions cause lasting changes in the brain’s stress system. This mechanism of action is thought to alter the brain’s reward pathways, worsen mood, increase cravings, and trap individuals in a continual cycle of problematic drinking and relapse vulnerability (Becker and Mulholland, 2014; Koob and Le Moal, 2001). During this cycle, the brain adapts in harmful ways, shifting motivation from drinking for pleasure to drinking to relieve negative feelings, and these long-term alterations of stress and reward systems may underlie compulsive drinking and high relapse risk that are seen in AUD (Koob, 2009; Koob and Le Moal, 1997).

The orexin (OX; or hypocretin) neuropeptide system originates from the hypothalamus (HYP) and projects throughout the brain (Peyron et al., 1998). It consists of two OXs, OXA and OXB, that are secondary products of the cleavage of a common precursor, prepro-OX (De Lecea et al., 1998; Sakurai et al., 1998). The OX system projects to many brain areas that are involved in stress and motivation, including the reward center that comprises the ventral tegmental area (VTA), nucleus accumbens (NAC), central nucleus of the amygdala, bed nucleus of the stria terminalis, and medial prefrontal cortex (mPFC; (Peyron et al., 1998). These neurons densely innervate the paraventricular nucleus of the thalamus (PVT), with the posterior PVT (pPVT) receiving the highest density of afferent OX terminals (Kirouac et al., 2005). Two OX receptors have been identified: OX1 receptor (which binds OXA) and OX2 receptor (which binds OXA and OXB with similar affinity; (Sakurai et al., 1998). Overall, OX promotes wakefulness, helps regulate sleep cycles (Chemelli et al., 1999; Hoyer and Jacobson, 2013; Inutsuka and Yamanaka, 2013; Krystal et al., 2013), influences stress responses (Berridge et al., 2010; Sargin, 2019), and enhances rewarding effects of both food and addictive drugs (Harris et al., 2005; Martin-Fardon and Boutrel, 2012; Matzeu and Martin-Fardon, 2020; Moorman and Aston-Jones, 2009; Thannickal et al., 2018).

Dynorphin (DYN) is an opioid peptide that is obtained from cleavage of the precursor pro-DYN, which primarily binds to κ-opioid receptors (KOPs; (Chavkin et al., 1982; Fallon and Leslie, 1986; Goldstein et al., 1979). Unlike OX, which is produced exclusively in the HYP, DYN is produced throughout the brain (Bali et al., 2014; Watson et al., 1982). Like OX, however, DYN influences various bodily functions and addictive properties of drugs (Bruchas et al., 2010; Wee and Koob, 2010). In humans, high levels of DYN-related genetic material (*PDYN* mRNA) are found in reward-related brain areas, including the striatum, NAC, and mPFC (Hurd, 1996). The DYN/KOP system is found throughout the brain (Watson et al., 1982) and plays a key role in negative emotional states that drive AUD (Koob, 2015). Most HYP neurons that produce OX also produce DYN (Chou et al., 2001; Li and van den Pol, 2006). Orexin and DYN are stored and released together from the same neurons, allowing them to interact in brain circuits that are involved in heightened drug seeking (Li and van den Pol, 2006; Muschamp et al., 2014). Despite being co-released, they have opposing effects. Orexin generally promotes reward seeking (like drug use), whereas DYN suppresses it. This is seen in such behaviors as cocaine self-administration, brain reward responses, and the firing rate of dopamine neurons in the VTA (Li and van den Pol, 2006; Muschamp et al., 2014). When applied together, their opposing actions balance out each other, causing no net change in VTA dopamine neuron activity (Muschamp et al., 2014). Blocking OX receptors in the VTA elevated brain stimulation reward thresholds, but blocking KOP receptors prevented this effect, demonstrating their functional interplay in reward (Muschamp et al., 2014). Similarly, in rats with a history of long-access (6 h/day) cocaine self-administration, DYN administration in the pPVT blocked the OX-induced reinstatement of extinguished cocaine-seeking behavior without altering the OX-induced reinstatement of extinguished sweetened condensed milk (SCM)-seeking behavior. These findings suggest that the ability of pPVT DYN to modulate OX-driven behavior may be specific to compulsive drug seeking and does not generalize to natural reward seeking (Matzeu et al., 2018a).

The PVT is part of dorsal midline thalamic nuclei and plays a significant role in the regulation of arousal/wakefulness, attention, awareness (Bentivoglio et al., 1991; Groenewegen and Berendse, 1994; Van der Werf et al., 2002), energy homeostasis, endocrine function, reward (Bhatnagar and Dallman, 1998; Kelley et al., 2005; Van der Werf et al., 2002), stress (Hsu et al., 2014), and drug seeking (Dayas et al., 2008; Martin-Fardon and Boutrel, 2012; Matzeu et al., 2017; Matzeu et al., 2018a; Matzeu et al., 2016; Matzeu and Martin-Fardon, 2020; Matzeu et al., 2015; Matzeu et al., 2014). High KOP expression is seen in the PVT (Mansour et al., 1996). Importantly, anatomical and functional differences between the anterior PVT (aPVT) and pPVT were suggested, such that the aPVT plays a role in arousal, and the pPVT plays a role in valence (e.g., (Barson et al., 2020). Moreover, a recent study showed that blocking OX transmission in the pPVT selectively prevented the stress-induced reinstatement of alcohol-seeking behavior at acute (8 h) abstinence selectively in alcohol-dependent rats (Matzeu and Martin-Fardon, 2020), suggesting that OX transmission in the pPVT is pivotal in the stress-induced reinstatement of alcohol-seeking behavior.

Emerging studies suggest that OX and DYN exert opposing effects in the VTA (Muschamp et al., 2014) and pPVT (Matzeu and Martin-Fardon, 2018) in the context of cocaine. However, critical knowledge gaps are (1) understanding how chronic alcohol (dys) regulates pPVT activity and impairs OX and DYN transmission and (2) understanding the role of OX and DYN transmission and their interaction in the pPVT during stress-induced alcohol-seeking behavior during alcohol abstinence. One hypothesis is that during abstinence following alcohol dependence, DYN’s ability to counteract OX-driven drug-seeking behavior may diminish, potentially exacerbating the vulnerability to relapse. Therefore, to investigate the role of OX and DYN transmission in the pPVT during stress-induced alcohol seeking during alcohol abstinence (∼3 weeks), using a multi-targeted *in vivo* pharmacological approach, the present study evaluated potential synergistic or opposite interactions among OX1/2 receptor and KOP signaling by co-administering OX receptor and KOP antagonists. Furthermore, this study evaluated whether, at the time of the reinstatement test, *Hcrt* and *Pdyn* mRNA expression in the HYP and *Hcrtr1*, *Hcrtr2*, and *Oprk1* mRNA expression in the pPVT were altered in alcohol-dependent rats and could help explaining the behavioral results.

## Methods

### Animals

A total of 60 male Wistar rats (Charles River Laboratories, Hollister, CA, United States), weighing 150–170 g upon arrival (∼6–7 weeks old), were pair-housed in a temperature- and humidity-controlled vivarium under a reverse 12 h/12 h light/dark cycle (lights off at 8:00 a.m.). Food and water were provided *ad libitum*. The rats were acclimated to the housing and handling conditions for 1 week before the experiments. Experimental manipulations, behavioral testing, and tissue collection were performed around 2:00 p.m., corresponding to Zeitgeber Time 18 (i.e., ZT18). All experimental procedures adhered to the National Institutes of Health Guide for the Care and Use of Laboratory Animals and Animal Research: Reporting *In Vivo* Experiments (ARRIVE) Guidelines (National Research Council, 2011; Percie du Sert et al., 2020) and were approved by The Scripps Research Institute’s Institutional Animal Care and Use Committee.

### Drugs

The dual OX receptor antagonist TCS1102 (Tocris Bioscience, Bristol, United Kingdom), KOP antagonist nor-binaltorphimine (norBNI; Abcam, Waltham, MA, United States), and TCS1102+norBNI combination were dissolved in 100% dimethylsulfoxide (DMSO; Sigma Aldrich, St. Louis, MO, United States). Control vehicle (VEH)-treated rats received 100% DMSO only. Pure DMSO was utilized because of TCS1102’s limited solubility in aqueous vehicles at the required concentration.

### Alcohol self-administration training

**FIGURE 1:**
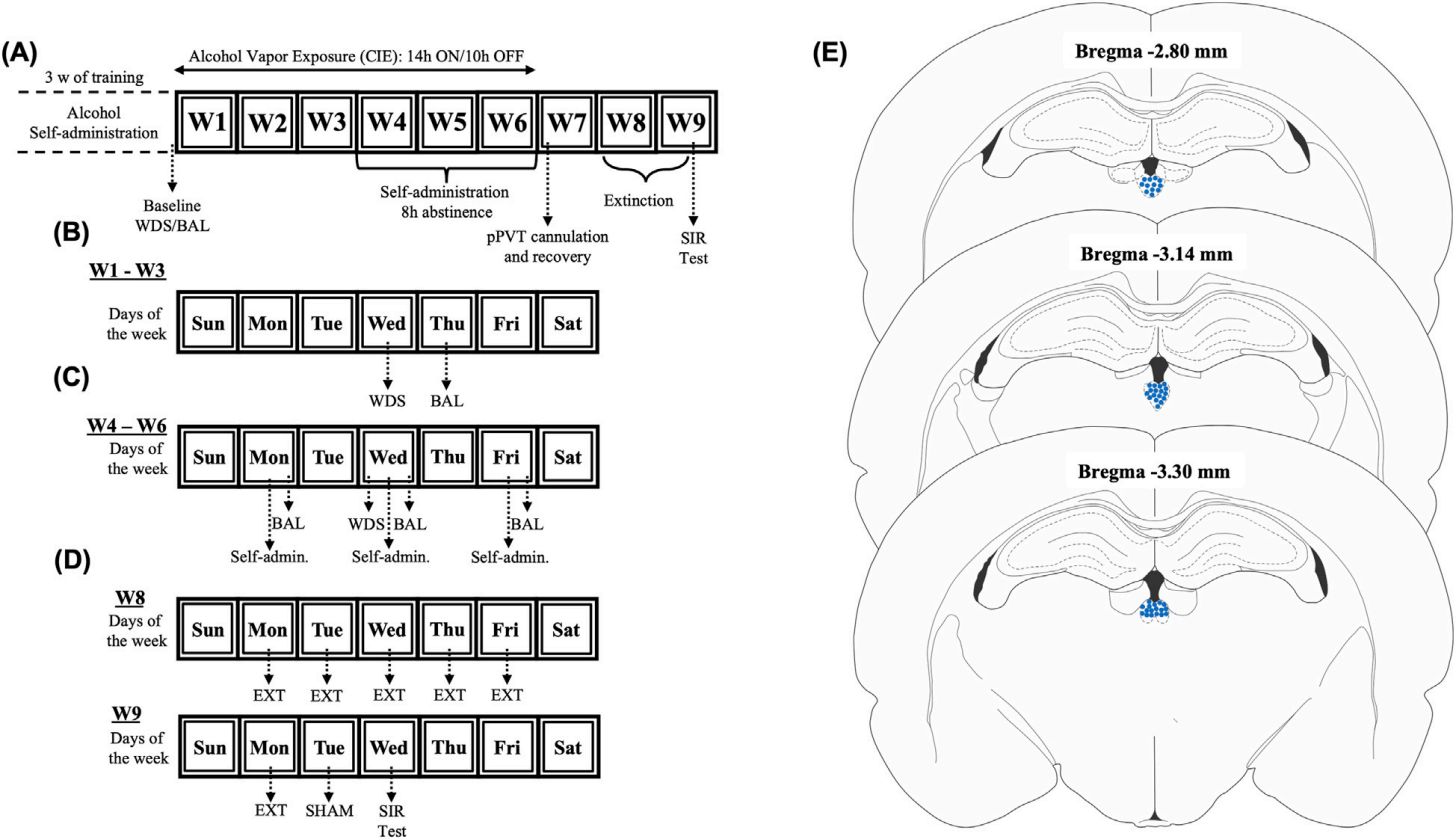
Experimental timeline. **(A)** Rats underwent 21 alcohol self-administration training sessions. Following training completion, baseline somatic withdrawal signs and blood alcohol levels were recorded. **(B)** Between weeks 1 and 3 of chronic intermittent alcohol vapor exposure, the rats were scored for somatic withdrawal signs 8 h after the vapor was turned OFF on Wednesday, and blood alcohol levels were recorded 30 min before the alcohol vapors were turned OFF on Thursdays. **(C)** The rats underwent self-administration sessions three times per week (Monday, Wednesday, and Friday) 8 h after the alcohol vapor was turned OFF between weeks 4 and 6 of alcohol vapor exposure. **(D)** Following 1 week of recovery from pPVT cannulation, the rats underwent extinction sessions twice daily. **(E)** Representation of injection sites. BAL, blood alcohol level; pPVT, posterior paraventricular nucleus of the thalamus; WDS, somatic withdrawal signs; W, week.

The rats underwent operant alcohol self-administration training as previously described (Flores-Ramirez et al., 2024; Flores-Ramirez et al., 2022a; Flores-Ramirez et al., 2023; Matzeu and Martin-Fardon, 2020). Briefly, after a 1-week acclimation period, daily 30-min self-administration sessions were conducted in standard operant chambers (Med Associates, St. Albans, VT, United States) under a fixed-ratio 1 schedule for 21 days (Figure 1A). Pressing the active (right) lever resulted in the delivery of 0.1 mL of 10% alcohol solution along with 0.5-s cue light activation. Pressing the inactive (left) lever was recorded but had no programmed outcomes. No fading procedures (e.g., saccharin/sucrose) were required to initiate voluntary alcohol consumption. Alcohol intake (g/kg) was calculated by normalizing active lever presses to daily body weight. Post-session reservoir checks were performed to confirm that all alcohol that was dispensed was consumed. Baseline self-administration levels were obtained by averaging the last three self-administration training sessions. After the completion of training, rats with comparable active-lever performance were randomly assigned to chronic intermittent ethanol vapor exposure and consequently stress-induced reinstatement or for quantitative polymerase chain reaction (qPCR) analyses.

### Chronic intermittent ethanol vapor exposure

Following self-administration training, the rats were divided into dependent (*n* = 24 chronic intermittent alcohol-exposed) and nondependent (*n* = 24 air-exposed) groups. Dependent rats underwent 6 weeks of daily 14-h alcohol vapor cycles that were interspersed with 10-h vapor OFF periods. Blood alcohol levels (BALs) were maintained at 150–250 mg% and measured weekly using a gas chromatography-headspace blood analyzer (Agilent Technologies, Santa Clara, CA, United States). Baseline BALs were measured immediately after the last alcohol self-administration training session (i.e., day 21; see Figure 1A). For 3 weeks, all rats remained undisturbed except for measuring BALs during the last 30 min of vapor exposure (on Thursday) and scoring somatic signs of withdrawal (at 8 h of abstinence) once weekly (on Wednesday; see Figure 1B). Withdrawal severity was assessed weekly by an observer who was blind to experimental conditions using a validated scale that scores somatic signs of withdrawal, including measures of ventromedial limb retraction, vocalization (i.e., irritability to touch), tail stiffness, abnormal gait, and body tremors. Each of these behaviors was assigned a score of 0–2, based on severity (0 = no signs, 1 = moderate signs, 2 = severe signs). To confirm dependence and assess withdrawal severity, the sum of the five scores (0–10) was used as a quantitative measure (Macey et al., 1996). Baseline withdrawal scores were measured before the last training session (day 21; see Figure 1A). During weeks 4-6, all rats (dependent and nondependent) completed tri-weekly (Monday, Wednesday, and Friday) fixed-ratio 1 alcohol self-administration sessions 8 h post-vapor termination when BALs were undetectable (Figure 1C). To further verify alcohol dependence, BALs were measured immediately after self-administration sessions at weeks 4, 5, and 6 of chronic intermittent alcohol vapor exposure. This approach was used because this model of alcohol dependence is well-known to induce motivational and somatic signs of withdrawal (Vendruscolo and Roberts, 2014). Air-exposed rats underwent identical procedures (i.e., BAL testing and withdrawal scoring) as dependent subjects.

### pPVT cannulation

After 6 weeks of alcohol vapor exposure, the rats were removed from the alcohol vapor chambers and started a ∼3-week abstinence period (Figure 1A). The rats were implanted with guide cannulas (23-gauge) that targeted the pPVT (stereotaxic coordinates: anterior/posterior, −3.3 mm; medial/lateral, ±2.72 mm; dorsal/ventral, −2.96 mm from dura, at a 25° angle; (Paxinos and Watson, 1998); under isoflurane anesthesia (1.0%–2.5%). Cannulas were positioned 3.5 mm above the final injection site. After a 7-day surgical recovery period, the rats started extinction training (see Figure 1A).

### Extinction training

Extinction sessions mirrored prior alcohol self-administration sessions, but alcohol delivery was withheld (Figure 1D). To acclimate the rats to footshock stress, they were placed in operant chambers 15 min before each session. Afterward, levers were extended to initiate the 30-min extinction session. Over 12 sessions (two 30-min sessions per day for 6 days), the rats learned to disassociate active lever pressing from alcohol reward. Twenty-four hours after the last extinction session, the rats received a sham microinjection for habituation to intracranial injections (Figure 1D), in which injectors were inserted into pPVT guide cannulas (Plastics One, Roanoke, VA, United States) for 2 min. The rats were then returned to their home cages for 2 min before a 15-min operant chamber habituation period. Finally, the levers were subsequently extended into the operant chambers to test the rats under extinction conditions.

### Stress-induced reinstatement

Twenty-four hours after the sham injection (Figure 1D), the rats received intra-pPVT microinjections of vehicle (VEH, DMSO), TCS1102 (15 μg/0.5 μL/side; (Dong et al., 2015; Flores-Ramirez et al., 2023; Hsiao et al., 2012; Matzeu and Martin-Fardon, 2020), norBNI (4 µg/0.5 µL; (Matzeu et al., 2018a; Whitfield et al., 2015), or their combination (TCS1102+norBNI) to test their effects on the stress-induced reinstatement of alcohol-seeking behavior. Because of norBNI’s long-lasting actions (Endoh et al., 1992), the rats in these groups were injected 24-h before combination treatment or reinstatement tests. Injections were delivered via a Harvard 22 syringe pump using injectors that extended 3.5 mm beyond the guide cannulas (0.5 μL/min over 1 min). The injectors remained in place for one additional minute post-infusion to ensure diffusion away from the injector tip. The rats were gently held during the procedure to minimize stress, and they were returned to their home cages for 2 min before being subjected to footshock stress (15 min; variable intermittent electric footshock, 0.5 mA; duration, 0.5 s; mean shock interval, 40 s; (Flores-Ramirez et al., 2022b; Flores-Ramirez et al., 2023; Martin-Fardon et al., 2000; Matzeu and Martin-Fardon, 2020; Sidhpura et al., 2010; Zhao et al., 2006). Two minutes post-stress, the levers were extended into the operant chamber, and responses were recorded for 30 min. To verify intra-pPVT injection sites, the rats were deeply anesthetized by CO_2_ inhalation, and their brains were rapidly harvested, snap-frozen in methyl butane, and cut into 40 μm sections using a cryostat (Leica CM3050S, Leica Biosystems Nusslich, Heidelberg, Germany). Using an adult rat brain atlas as a reference (Paxinos and Watson, 1998), the injection sites were verified, and off-target cannulations were excluded from the study (Figure 1E).

### Measures of Hcrt, Pdyn, Hcrtr1, Hcrtr2, and Oprk1 mRNA expression by qPCR

A separate group of rats (*n* = 8) for gene expression analysis underwent the same behavioral procedures, including alcohol dependence induction, but did not receive pPVT cannulation or injections or undergo reinstatement testing. The rats were euthanized via CO_2_ inhalation (3–7 L/min) 24 h after the last extinction session, corresponding to the time when the behavioral group of rats was tested for stress-induced reinstatement. Brains were rapidly harvested, snap-frozen in methylbutane, and stored at −80 °C. An additional control group that was experimentally naive to all conditions (*n* = 4) was also prepared, and their brains were processed similarly. Brains were dissected into serial coronal sections and the pPVT, as well as whole HYP, was collected using tissue punches (World Precision Instruments, Sarasota, FL, United States). RNA was isolated using Zymo Research RNA concentrator-5 kits (Irvine, CA, United States), quantified using a NanoDrop 2000c spectrophotometer (Thermo Fisher Scientific, Waltham, MA, United States), and reverse-transcribed to cDNA using 5X mix, iScript, reverse transcription, and Supermix for real-time qPCR (RT-qPCR) with the CFX 384 Real-Time System (Bio-Rad, Hercules, CA, United States). To amplify cDNA, SYBR, iTap Universal SYBR, and Green Supermix were used and analyzed using duplicate samples. Cycle threshold (Ct) values were determined, and changes in gene expression were assessed using the ΔΔCt method with β-actin as the housekeeping reference gene. The forward and reverse primer sequences of the antisense oligonucleotides were the following: *β-actin* (forward, 5′-ATC TGG CAC ACC TTC-3’; reverse, 5′-AGC CAG GTC CAG ACG CA-3′), *Hcrtr1* (forward, 5′-CCC TCA ACT CCA GTC CTA GC-3’; reverse, 5′-CAG GGA GGG CCT ATA ATT GA-3′), *Hcrtr2* (forward, 5′-CCA TGT TGG GGT GCT TA-3’; reverse, 5′-TCC CCC TCT CAT AAA CTT GG-3′), *Oprk1* (forward, 5′-CCA AAG TCA GGG AAG ATG TGG A-3’; reverse, 5′-TCA AGC GCA GGA TCA TCA GG-3′), *Pdyn* (forward, 5′-CCA GGC TAT GCA GCA GAA GA-3’; reverse, 5′-GCT GTC AGC GTC TTC GTC TA-3′), and *Hcrt* (forward, 5′-GCC CTC TCT ACG AAC TGT TG-3’; reverse, 5′-CGA GGA GAG GGG AAA GTT AG-3′).

### Statistical analysis

Alcohol self-administration was analyzed using two-way repeated-measures analysis of variance (ANOVA), with time and alcohol dependence as factors. Self-administration data during chronic alcohol vapor exposure (baseline vs. weeks 4–6) and BALs after self-administration sessions were also analyzed using two-way repeated-measures ANOVA, with time and alcohol dependence as factors. Chronic intermittent alcohol vapor exposure’s effect on somatic withdrawal signs was analyzed using a Friedman test, followed by Dunn’s tests. Effects of TCS1102, norBNI, and their combination on active lever responses during the stress-induced reinstatement of alcohol-seeking behavior were analyzed using a two-way repeated measures ANOVA, with alcohol dependence and treatment as sources of variance. The gene expression data were analyzed using one-way ANOVA. Significant interactions and main effects were followed by the Bonferroni *post hoc* test for all ANOVAs. The data are expressed as the mean +SEM. Values of *p* < 0.05 were considered statistically significant. The analyses were performed using GraphPad Prism 10.4.2 software.

## Results

### Alcohol self-administration and escalation

**FIGURE 2:**
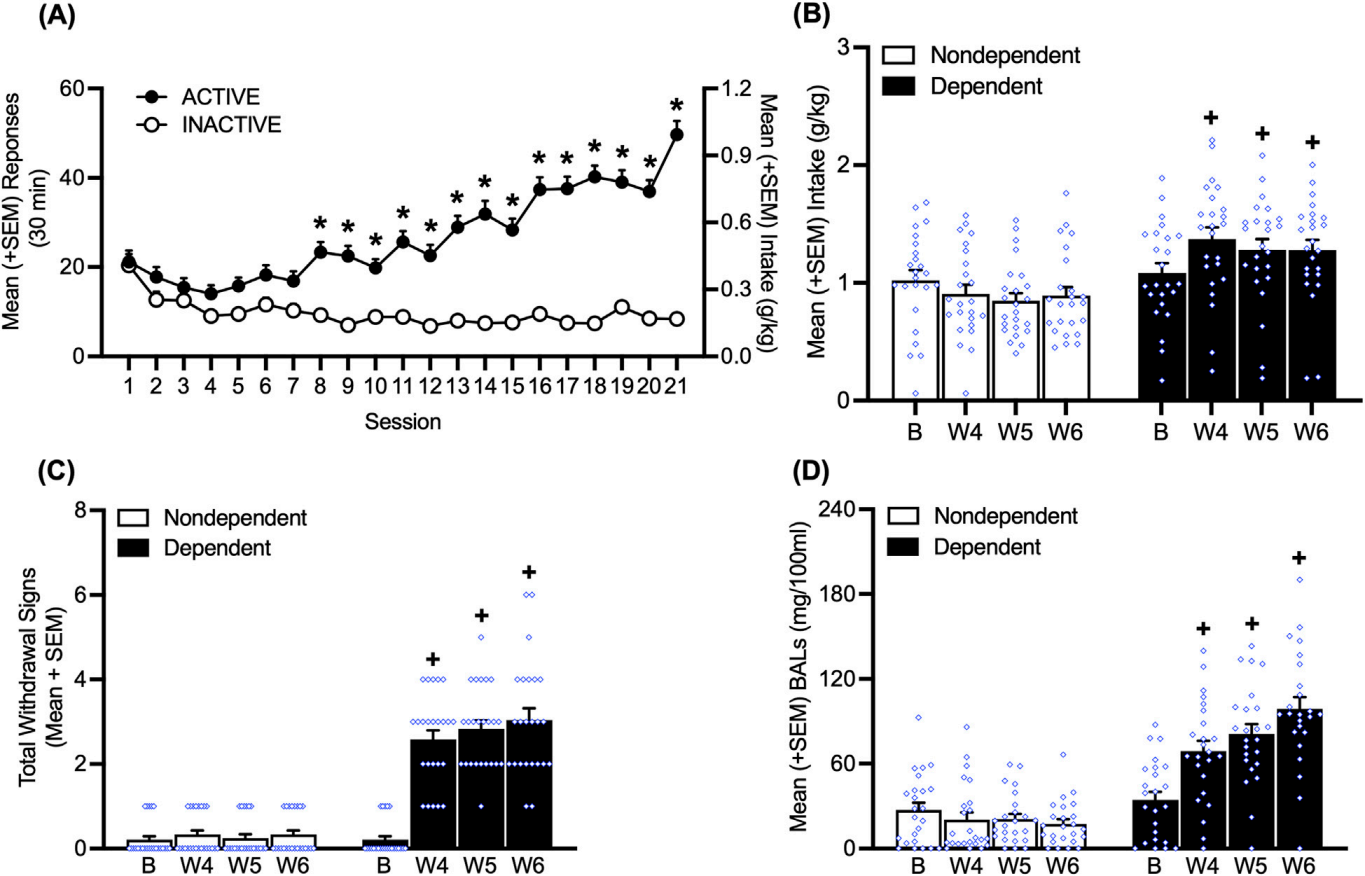
Time course of alcohol self-administration over 21 sessions of training and the escalation of drinking during weeks 4, 5, and 6 of chronic intermittent alcohol vapor exposure. **(A)** Starting in session 8, the rats responded more on the active lever. **(B)** At weeks 4, 5, and 6 of chronic intermittent alcohol vapor exposure, dependent rats exhibited an increase in alcohol drinking. **(C)** During acute abstinence, an increase in somatic withdrawal signs (WDS) was observed in dependent rats at weeks 4, 5, and 6 of chronic intermittent alcohol vapor exposure. **(D)** After the self-administration sessions at weeks 4, 5, and 6 of chronic intermittent alcohol vapor exposure, alcohol-dependent rats had higher blood alcohol levels than nondependent rats. *p < 0.05, vs. inactive lever; ^+^ *p* < 0.05, vs. respective baseline. B, baseline; BAL, blood alcohol level; W, week.

Rats (*n* = 48) acquired alcohol self-administration over 21 sessions of training (30 min/day; two-way repeated-measures ANOVA; time: *F*_20,1880_ = 18.07, *p* < 0.05; lever: *F*_1,94_ = 107.47, *p* < 0.05; time × lever interaction: *F*_20,1880_ = 25.98, *p* < 0.05; Figure 2A). The Bonferroni *post hoc* tests confirmed that the number of responses at the active lever was significantly higher than responses at the inactive lever starting at session 8 (*p* < 0.05; Figure 2A).

During weeks 4, 5, and 6 of chronic intermittent alcohol vapor exposure, alcohol-dependent rats exhibited the escalation of alcohol intake, a measure that was obtained by averaging the intake data that were recorded on Monday, Wednesday, and Friday of that week (*p* < 0.05, Bonferroni *post hoc* test vs. baseline following two-way repeated-measures ANOVA; alcohol dependence: *F*_1,46_ = 10.26, *p* < 0.05; time × alcohol dependence interaction: *F*_3,138_ = 8.42, *p* < 0.05; Figure 2B). During weeks 4, 5, and 6, alcohol-dependent rats exhibited significantly higher somatic withdrawal signs at an acute abstinence point (8 h after vapors were off; *p* < 0.05, Dunn’s test vs. baseline following Friedman non-parametric test: χ^2^ (7) = 135.4, *p* < 0.05; Figure 2C). Alcohol-dependent rats had higher BALs after the self-administration sessions at weeks 4, 5, and 6 (*p* < 0.05, Bonferroni *post hoc* test vs. baseline following two-way repeated-measures ANOVA; time: *F*_3,138_ = 11.60, *p* < 0.05; alcohol dependence: *F*_1,46_ = 68.75, *p* < 0.05; time × alcohol dependence interaction: *F*_3,138_ = 21.05, *p* < 0.05; Figure 2D).

### Extinction and stress-induced reinstatement

**FIGURE 3:**
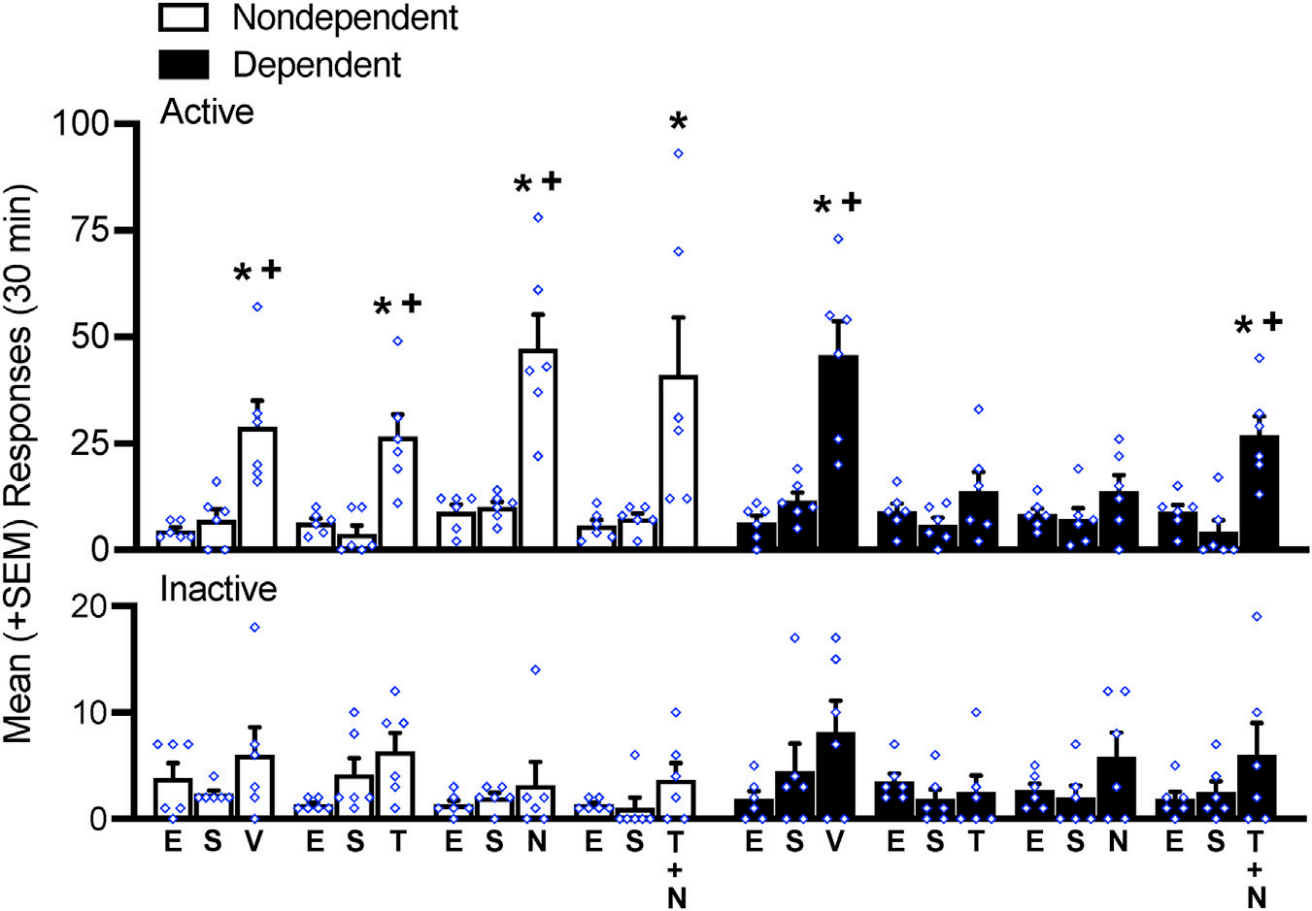
Intermittent footshock stress precipitated alcohol-seeking behavior in rats that received vehicle (V) in both dependent and nondependent groups. In dependent rats, the dual OX receptor antagonist TCS1102 and KOP antagonist norBNI decreased the stress-induced reinstatement of alcohol-seeking behavior, an effect that was not observed when concomitant TCS1102 and norBNI were administered. No differences in inactive lever responses were observed. *p < 0.05, vs. respective extinction (E); ^+^ *p* < 0.05, vs. respective sham (S). E, extinction; S, sham; V, vehicle; N, norBNI; T, TCS1102.

Over 12 sessions of extinction training, the number of responses at the active lever gradually decreased until the total number of responses on the active lever was indistinguishable from the number of responses at the inactive lever (data not shown), similar to earlier studies (e.g., (Flores-Ramirez et al., 2022b; Flores-Ramirez et al., 2023). Following extinction training (EXT) and sham injections (SHAM), nondependent and dependent rats (N = 48) were assigned to vehicle (n = 6), TCS (n = 6), norBNI (n = 6), or the combination (n = 6) per their respective history of dependence. Sham injections did not reinstate or suppress any behavior, as displayed by the rat’s performance at the active lever (*p* > 0.05, Bonferroni *post-hoc* test EXT vs. SHAM following a two-way repeated measures ANOVA; treatment: *F*_2,80_ = 89.78, *p* < 0.05; alcohol dependence: *F*_7,40_ = 3.10, *p* < 0.05; treatment × alcohol dependence interaction: *F*_14,80_ = 3.49, *p* < 0.05; Figure 3). Responses on the inactive lever remained at the level of extinction in both dependent and nondependent rats (*p* > 0.05, Bonferroni *post-hoc* test EXT vs. SHAM following two-way repeated measures ANOVA; treatment: *F*_2,80_ = 8.19, *p* < 0.05; alcohol dependence: *F*_7,40_ = 1.25, *p* > 0.05; treatment × alcohol dependence interaction: *F*_14,80_ = 0.60, *p* > 0.05; Figure 3).

Stress precipitated the reinstatement of alcohol-seeking behavior in both nondependent and dependent rats under VEH conditions (Figure 3). In nondependent rats, the administration of TCS1102 (*p* < 0.05, Bonferroni *post-hoc* tests vs. EXT and *p* < 0.05, Bonferroni *post-hoc* tests vs. SHAM), norBNI (*p* < 0.05, Bonferroni *post-hoc* tests vs. EXT and *p* < 0.05, Bonferroni *post-hoc* tests vs. SHAM), and the TCS1102+norBNI combination (*p* < 0.05, Bonferroni *post-hoc* tests vs. EXT) did not modify reinstatement (*post-hoc* tests following a two-way repeated measures ANOVA; treatment: *F*_2,80_ = 89.78, *p* < 0.05; alcohol dependence: *F*_7,40_ = 3.10, *p* < 0.05; treatment × alcohol dependence interaction: *F*_14,80_ = 3.49, *p* < 0.05; Figure 3). In dependent rats, the administration of TCS1102 and norBNI significantly reduced the stress-induced reinstatement of alcohol-seeking behavior (*p* > 0.05, Bonferroni *post-hoc* tests vs. EXT and *p* > 0.05, Bonferroni *post-hoc* tests vs. SHAM following a repeated measures ANOVA; treatment: *F*_2,80_ = 89.78, *p* < 0.05; alcohol dependence: *F*_7,40_ = 3.10, *p* < 0.05; treatment × alcohol dependence interaction: *F*_14,80_ = 3.49, *p* < 0.05; Figure 3) but not when they were administered together (*p* < 0.05, Bonferroni *post-hoc* tests vs. EXT and *p* < 0.05, Bonferroni *post-hoc* tests vs. SHAM following a two-way repeated measures ANOVA; Figure 3). No differences in inactive lever responses were observed, regardless of a history of alcohol-dependence and treatment condition (*p* > 0.05, Bonferroni *post-hoc* test EXT vs. SHAM following two-way repeated measures ANOVA; treatment: *F*_2,80_ = 8.19, *p* < 0.05; alcohol dependence: *F*_7,40_ = 1.25, *p* > 0.05; treatment × alcohol dependence interaction: *F*_14,80_ = 0.60, *p* > 0.05; Figure 3).

### Measures of Hcrt, Pdyn, Hcrtr1, Hcrtr2, and Oprk1 mRNA expression by qPCR

**FIGURE 4:**
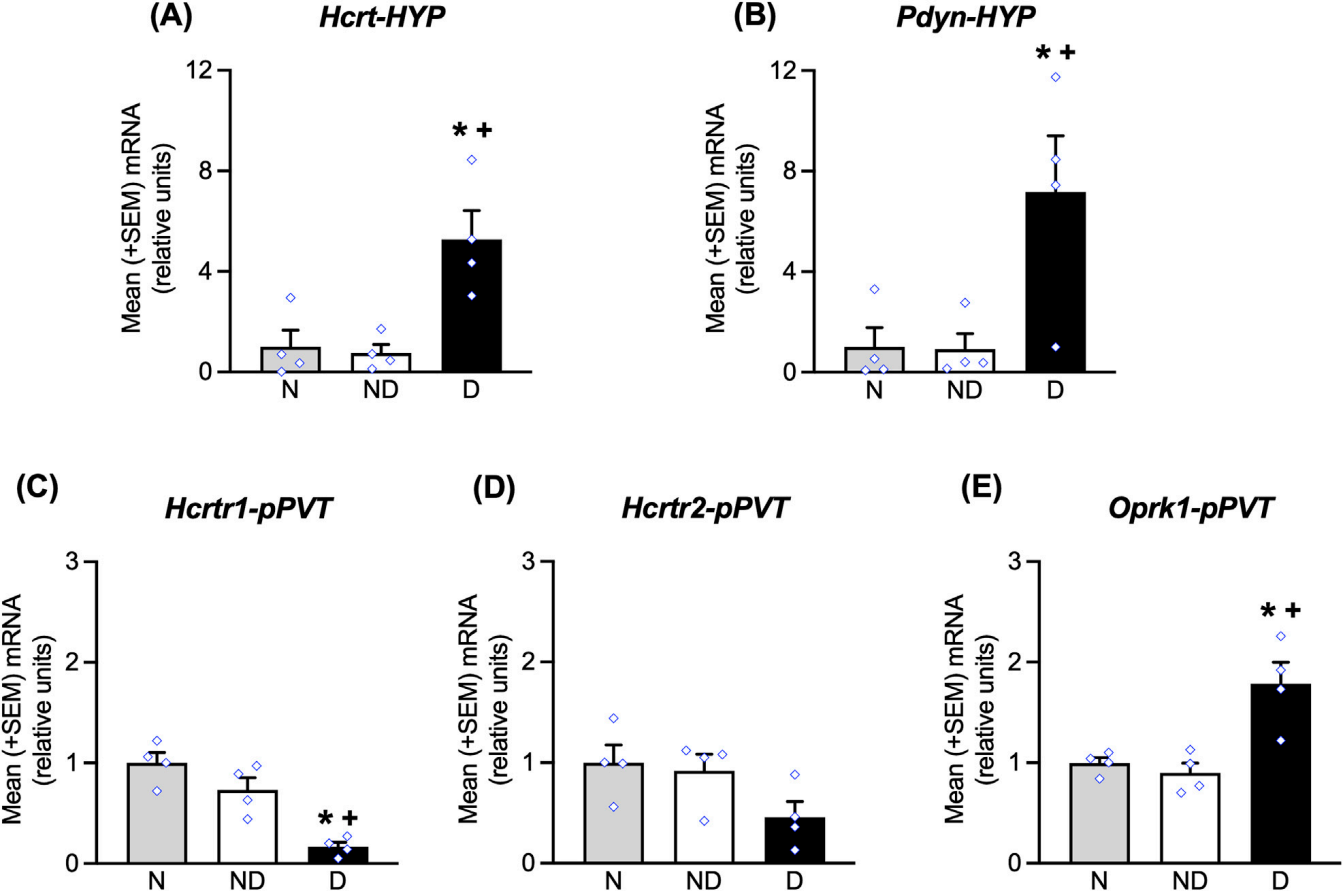
mRNA expression of *Hcrt* and *Pdyn* in the HYP and *Hcrtr1*/*2* and *Oprk1* in the pPVT during late abstinence (∼3 weeks), corresponding to the time when the stress-induced reinstatement tests were conducted. At the time of testing, in the HYP, increases in *Hcrt* **(A)** and *Pdyn* **(B)** mRNA expression were observed. In the pPVT, decreases in *Hcrtr1* **(C)**, no changes in *Hcrtr2* **(D)** and increases in *Oprk1* **(E)** mRNA expression were measured. *p < 0.05, vs. naive; ^+^ *p* < 0.05, vs. nondependent. N, naive; ND, nondependent; D, dependent.

The group of rats that were used for this experiment acquired alcohol self-administration training, increased their alcohol intake during dependence induction, and exhibited the extinction of alcohol-seeking behavior similarly to the behavioral groups (data not shown). Analyses of mRNA expression in the HYP showed that at ∼3 weeks of abstinence, significant increases in *Hcrt* mRNA expression (Bonferroni *post-hoc* tests, *p* < 0.05, vs. naive; *p* < 0.05, vs. nondependent following one-way ANOVA: *F*_2,9_ = 10.24, *p* < 0.05; Figure 4A) and *Pdyn* mRNA expression (Bonferroni *post-hoc* tests, *p* < 0.05, vs. naive; *p* < 0.05, vs. nondependent following one-way ANOVA: *F*_2,9_ = 6.37, *p* < 0.05; Figure 4B) were observed. In the pPVT, the analyses showed a significant decrease in *Hcrtr1* mRNA expression (Bonferroni *post-hoc* tests, *p* < 0.05, vs. naive; *p* < 0.05, vs. nondependent following one-way ANOVA: *F*_2,9_ = 19.61, *p* < 0.05; Figure 4C), no significant changes in *Hcrtr2* mRNA expression (*p* > 0.05; Figure 4D), and an increase in *Oprk1* mRNA expression (Bonferroni *post-hoc* tests, *p* < 0.05, vs. naive; *p* < 0.05, vs. nondependent following one-way ANOVA: *F*_2,21_ = 11.76, *p* < 0.05; Figure 4E).

## Discussion

The present study examined the participation of pPVT OX and DYN transmission, specifically the OX receptor/KOP signaling interaction, during the stress-induced reinstatement of alcohol-seeking behavior in alcohol-dependent rats during abstinence. We also assessed molecular alterations of HYP *Hcrt* and *Pdyn* mRNA expression and pPVT *Hcrtr1*, *Hcrtr2*, and *Oprk1* mRNA expression during abstinence. Alcohol-dependent rats exhibited the escalation of alcohol self-administration under chronic vapor exposure conditions, consistent with previous findings (Flores-Ramirez et al., 2024; Flores-Ramirez et al., 2022a; Flores-Ramirez et al., 2023; Matzeu et al., 2018a; Matzeu and Martin-Fardon, 2020). Parallelling prior work (e.g., (Flores-Ramirez et al., 2024; Flores-Ramirez et al., 2022a; Le et al., 1999; Martin-Fardon et al., 2000), intermittent unpredictable footshock stress effectively reinstated alcohol seeking. Notably, in dependent rats, blocking OX1/2 receptor or KOP signaling in the pPVT prevented the stress-induced reinstatement of alcohol-seeking behavior, which was not observed when OX1/2 receptor and KOP signaling were concomitantly blocked (i.e., TCS1102 + norBNI co-administration). Post-extinction changes in *Hcrt* and *Pdyn* mRNA expression in the HYP and *Hcrtr1* and *Oprk1* mRNA expression in the pPVT indicated persistent alterations of OX receptor and KOP signaling. These outcomes underscore, for the first time, the alcohol dependence-induced dysregulation of OX and DYN neurotransmission and their functional interplay during stress-driven relapse to alcohol seeking.

Preclinical research has demonstrated that alcohol-dependent rats develop the escalation of alcohol consumption alongside somatic and motivational withdrawal signs within 6–8 h of abstinence (Flores-Ramirez et al., 2024; Flores-Ramirez et al., 2022a; Flores-Ramirez et al., 2023; Matzeu et al., 2018a; Vendruscolo and Roberts, 2014). In the present study, rats that were subjected to 6 weeks of chronic intermittent alcohol vapor exposure similarly manifested increases in alcohol self-administration, withdrawal symptoms, and BALs following self-administration sessions. These results support the vapor exposure model’s ability to induce behavioral and physiological indicators of dependence. The underlying mechanism likely involves dysregulation within neural circuits that are responsible for inhibitory control, reward valuation, and stress reactivity. This dysregulation could play a role in hyperkatifeia—a negative emotional state during withdrawal where alcohol seeking is motivated by relief from distress, particularly under stressful conditions (Koob, 2014).

The intra-pPVT infusion of TCS1102 blocked the stress-induced reinstatement of alcohol seeking in alcohol-dependent rats, echoing previous findings that showed that inhibiting OX receptor signaling selectively reduces alcohol-directed behaviors in dependent rats. For example, subcutaneous administration of the OX2 receptor antagonist JNJ-10397049 reduced alcohol intake without altering saccharin consumption in male Wistar rats (Shoblock et al., 2011), and an infusion of the OX2 receptor antagonist TCSOX229 in the NAC core but not shell decreased alcohol self-administration without affecting conditioned reinstatement in male inbred alcohol-preferring (iP) rats (Brown et al., 2013). Similarly, OX1 receptor blockade lowered progressive-ratio responding for alcohol in male iP rats (Jupp et al., 2011) and selectively suppressed alcohol but not SuperSac (a highly palatable sweet solution) seeking in male Wistar rats (Martin-Fardon and Weiss, 2014). More recently, another study showed that TCS1102 administration in the pPVT selectively prevented the stress-induced the reinstatement of alcohol-seeking behavior at acute (8 h) abstinence (Matzeu and Martin-Fardon, 2020), suggesting that this selectivity likely reflects the dependence-driven dysregulation of OX signaling in the pPVT, given that OX receptor antagonists exert stronger effects in dependent or high-preferring rats (Flores-Ramirez et al., 2022a; Flores-Ramirez et al., 2023; Matzeu and Martin-Fardon, 2020; Moorman and Aston-Jones, 2009; Moorman et al., 2017).

The present findings align with earlier work that showed that KOP antagonists did not affect baseline alcohol drinking in nondependent subjects but attenuated escalated intake and seeking in highly motivated states that were exhibited by dependent subjects (Domi et al., 2018; Flores-Ramirez et al., 2024; Walker and Koob, 2008; Walker et al., 2011). This is also consistent with an earlier report that showed that systemic or intracerebroventricular norBNI administration blocked withdrawal-driven drinking in alcohol-dependent male Wistar rats (Walker and Koob, 2008; Walker et al., 2011), and oral administration of the KOP antagonist LY2444296 selectively reduced alcohol consumption in both male and female dependent rats (Flores-Ramirez et al., 2024). Similarly, oral administration of the KOP antagonist CERC-501 lowered free-access alcohol intake in alcohol-preferring P rats (Rorick-Kehn et al., 2014). This greater sensitivity to KOP antagonism in alcohol-dependent rats supports a model in which repeated alcohol exposure upregulates KOP signaling, producing stress and dysphoria that drive the negative reinforcement-mediated reinstatement of alcohol seeking (Wee and Koob, 2010). Thus, in subjects that have greater motivation to seek and take alcohol, KOP signaling may be compromised, magnifying the incentive value of alcohol via negative reinforcement systems.

Importantly, the inhibitory effects of TCS1102 and norBNI on reinstatement when they were administered alone, which was observable only in alcohol-dependent rats, were modulated when they were co-administered. These findings clearly point to functional crosstalk between OX1/2 receptor and KOP signaling in the pPVT during the stress-induced reinstatement of alcohol-seeking behavior post-dependence. Previous work has shown that systemic or intra-VTA SB334867 administration elevates intracranial self-stimulation thresholds, an effect that was reversed by norBNI, demonstrating the involvement of KOPs and their interaction with OX receptor signaling. In mouse VTA slices, most dopaminergic neurons (65.4%) co-respond to OX and DYN without net changes in firing, whereas certain subsets favor OX (16.9%) or DYN (7.7%; (Muschamp et al., 2014). In the lateral HYP, exogenous OX and DYN exert opposing effects on neuronal excitability (Li and van den Pol, 2006), and the optogenetic activation of OX/DYN neurons bidirectionally attenuated VTA dopamine firing, whereas OX1 receptor blockade reduced excitation, and KOP antagonism reduced inhibition, suggesting peptide co-release into the VTA (Mohammadkhani et al., 2024). Finally, in the pPVT, OX was shown to increase glutamate release, and DYN co-infusion suppressed OX-driven glutamate release (Matzeu et al., 2018a). Moreover, the same study reported that DYN administration in the pPVT in rats with a history of chronic excessive cocaine intake blocked the OX-induced reinstatement of extinguished cocaine-seeking behavior without altering the OX-induced reinstatement of extinguished SCM-seeking behavior (Matzeu et al., 2018a). Together with the extant literature, the present results confirm that region-specific peptide levels, receptor localization, and intracellular pathways shape OX–DYN interactions, and this interplay influences the stress-induced reinstatement of alcohol-seeking behavior in dependent rats.

At the time when the stress-induced reinstatement was assessed (∼3 weeks of abstinence), a significant increase in *Hcrt* mRNA expression in the HYP and a decrease in *Hcrtr1* mRNA expression in the pPVT were observed. Previous work reported increases in *Hcrt* mRNA expression in the lateral HYP in genetically selected iP rats (Lawrence et al., 2006) and an increase in *Hcrtr2* expression in the aPVT (Barson et al., 2015). Furthermore, earlier observations from our laboratory showed that alcohol-dependent rats in acute (8 h) abstinence exhibited an increase in *Hcrt* mRNA expression in the HYP and an increase in *Hcrtr1/2* mRNA expression in both the pPVT and infralimbic cortex (Flores-Ramirez et al., 2023; Matzeu and Martin-Fardon, 2020). Therefore, the results of the present study suggest that chronic alcohol exposure not only affects OX transmission at acute abstinence but also well into abstinence (i.e., ∼3 weeks), potentially via the dysregulation of pPVT OX1 receptors, thereby promoting escalation of drinking and relapse vulnerability. Furthermore, the present study found increases in *Pdyn* and *Oprk1* mRNA expression in the HYP and pPVT, respectively. Notably, repeated cocaine administration increased *Pdyn* mRNA expression and decreased KOP expression in the NAC and striatum up to 48 h after cocaine exposure in male Wistar rats (Turchan et al., 1998). Withdrawal from alcohol (Lindholm et al., 2000) was also shown to be associated with an increase in *Pdyn* mRNA expression in male Sprague-Dawley rats. Together with the present findings, this suggests that alcohol dependence dysregulates the OX/OX receptor and DYN/KOP systems, potentially compromising and promoting the incentive to drink alcohol through negative reinforcement mechanisms, thereby increasing the vulnerability to relapse. It is important to note, however, that mRNA elevation alone does not prove functional coupling, receptor localization, or net circuit-level outcome (Lee et al., 2003; Mehra, Lee and Hatzimanikatis, 2003). Therefore, future studies will be needed to address the exact molecular mechanism driving the behavioral effects observed in the present study.

One key limitation of the present study was the exclusion of female subjects, which restricts generalizability of the findings. Indeed, prior research documented sex differences in alcohol intake and preference (Blanchard et al., 1993; Li and Lumeng, 1984; Walker et al., 2008), sensitivity to alcohol’s rewarding and aversive effects (Torres et al., 2014), and differences in BALs after self-administration and somatic withdrawal signs during intermittent alcohol vapor exposure (Matzeu et al., 2018b). Furthermore, OXs have been found to mediate sex differences in stress responsivity, and there are also sex-mediated differences in KOP signaling and function (Chartoff and Mavrikaki, 2015; Ma et al., 2023). Another limitation is that the qPCR analyses were conducted in rats that did not undergo the stress-induced reinstatement of alcohol seeking and did not receive any injections in the pPVT. Assessing changes in mRNA in rats that are concomitantly exposed to dependence, stress, and pharmacological manipulations would certainly be an interesting line of investigation. The rationalization for our approach was to obtain a clearer picture of molecular changes that are induced specifically by alcohol dependence itself and set the stage for OX and DYN to modulate reinstatement-like behavior. The potential toxicity for the use of pure DMSO as vehicle cannot be excluded and could be another limitation of the study. This vehicle choice was driven by solubility constraints of the OX receptor antagonist and its successful use in previous studies from our group (e.g., Flores-Ramirez et al., 2022a; Flores-Ramirez et al., 2023; Illenberger et al., 2024; Matzeu and Martin-Fardon, 2020). It is important to note that because all groups received the same concentration of DMSO and no behavioral nonspecific side effects nor tissue damage were observed, we are confident that the use of pure DMSO in the present study did not introduce any behavioral confound. Ultimately, although the OX/DYN interaction is observable via the behavioral pharmacology results in the present study, the exact molecular mechanisms that drive this interaction are currently unknown. Thus, future studies should assess the actual mechanisms that drive these effects and the unique contribution of alcohol dependence in shaping these mechanisms.

Overall, the present study demonstrated for the first time that the OX/DYN functional interaction in the pPVT played a significant role in stress-induced relapse in alcohol-dependent subjects. Chronic alcohol use appears to dysregulate pPVT functionality via maladaptive changes in OX and DYN transmission, facilitating the shift from controlled to compulsive drinking. This may highlight the need for multi-target therapies that restore pPVT balance and function to curb stress-triggered craving and relapse in subjects that undergo self-imposed abstinence.

## Data Availability

The raw data supporting the conclusions of this article will be made available by the authors, without undue reservation.
